# Inappropriate empiric antifungal therapy for candidemia in the ICU and hospital resource utilization: a retrospective cohort study

**DOI:** 10.1186/1471-2334-10-150

**Published:** 2010-06-03

**Authors:** Marya D Zilberberg, Marin H Kollef, Heather Arnold, Andrew Labelle, Scott T Micek, Smita Kothari, Andrew F Shorr

**Affiliations:** 1 EviMed Research Group, LLC, PO Box 303, Goshen, MA 01032, USA; 2 School of Public Health and Health Sciences, University of Massachusetts, 715 North Pleasant Street, Amherst, MA 01003, USA; 3 Department of Medicine, Washington University School of Medicine, 660 Euclid Avenue, St. Louis, MO 63110, USA; 4 Health Economics and Outcomes Research, Astellas Pharma US, Inc., 3 Parkway North, Deerfield, IL 60015, USA; 5 Division of Pulmonary and Critical Care, Washington Hospital Center, 100 Irving Street NW, Washington, DC 20010, USA

## Abstract

**Background:**

Candida represents the most common cause of invasive fungal disease, and candidal blood stream infections (CBSI) are prevalent in the ICU. Inappropriate antifungal therapy (IAT) is known to increase a patient's risk for death. We hypothesized that in an ICU cohort it would also adversely affect resource utilization.

**Methods:**

We retrospectively identified all patients with candidemia on or before hospital day 14 and requiring an ICU stay at Barnes-Jewish Hospital between 2004 and 2007. Hospital length of stay following culture-proven onset of CBSI (post-CBSI HLOS) was primary and hospital costs secondary endpoints. IAT was defined as treatment delay of ≥24 hours from candidemia onset or inadequate dose of antifungal agent active against the pathogen. We developed generalized linear models (GLM) to assess independent impact of inappropriate therapy on LOS and costs.

**Results:**

Ninety patients met inclusion criteria. IAT was frequent (88.9%). In the IAT group antifungal delay ≥24 hours occurred in 95.0% and inappropriate dosage in 26.3%. Unadjusted hospital mortality was greater among IAT (28.8%) than non-IAT (0%) patients, p = 0.059. Both crude post-CBSI HLOS (18.4 ± 17.0 vs. 10.7 ± 9.4, p = 0.062) and total costs ($66,584 ± $49,120 vs. $33,526 ± $27,244, p = 0.006) were higher in IAT than in non-IAT. In GLMs adjusting for confounders IAT-attributable excess post-CBSI HLOS was 7.7 days (95% CI 0.6-13.5) and attributable total costs were $13,398 (95% CI $1,060-$26,736).

**Conclusions:**

IAT of CBSI, such as delays and incorrect dosing, occurs commonly. In addition to its adverse impact on clinical outcomes, IAT results in substantial prolongation of hospital LOS and increase in hospital costs. Efforts to enhance rates of appropriate therapy for candidemia may improve resource use.

## Background

*Candida sp. *is the most frequent cause of invasive fungal disease in hospitalized patients [1]. The annual incidence of hospitalizations in the US associated with candidal blood stream infections (BSI) has risen by 50% between 2000 and 2005 [2]. In addition, Candida now accounts for 12% of all hospital-acquired BSIs [3]. Concurrent with this absolute growth in the burden of candidemia, healthcare institutions have also witnessed an increase in the proportion of infections caused by azole-resistant *Candida *species, such as *C. glabrata *and *C. krusei *[4,5]. These microbiologic shifts have challenged clinicians' ability to predict the spectrum of necessary coverage in an empiric regimen aimed at a suspected *Candida *infection.

At the same time, a growing body of evidence points to an association between inappropriate early selection of an antifungal agent and worsened clinical outcomes [6-8]. Most studies indicate that failing to administer appropriate coverage, defined as a drug that is *in vitro *active against the culprit pathogen, within 24 hours of infection onset, independently raises the patient's probability of death by a factor of two or more.

There is therefore little doubt that early recognition and knowledge of local patterns of candidal resistance can aid efforts to select appropriate coverage, which in turn should improve clinical outcomes. What is unclear is the impact of inappropriate therapy on economic parameters, namely length (LOS) and costs of hospitalizations. While on the one hand, the LOS may be reduced due to an early mortality associated with inappropriate and/or delayed therapy, it is also possible that such therapy may actually prolong the LOS, and thus the costs, of a hospitalization before resulting in death. We hypothesized that the latter is the case, and conducted a study to examine this hypothesis.

## Methods

We conducted a retrospective single-center cohort study at the Barnes-Jewish Hospital (BJH), St. Louis, MO. The Washington University School of Medicine Human Studies Committee approved the study, and informed consent was waved. BJH is a large (1,200 beds) urban academic medical center serving an inner-city population. Given that the majority of candidemia patients are found in the ICU, we included all patients with culture-proven candidemia occurring on or before hospital day 14 and requiring an ICU stay between January 1, 2004 and December 31, 2007. At the BJH certain BMT and solid organ transplant patients receive antifungal prophylaxis, with the duration and agent dependent on how far out they are from transplant and what organ or type of stem cell donor they have. Overall, however, for standard ICU patients, antifungal prophylaxis is not provided. Empiric coverage is not standardized. For those patients at high risk for fungemia, antifungal therapy is initiated with broad-spectrum antifungal therapy at the onset of signs and symptoms of infection. Those with a lower risk for fungemia are not started on therapy until they remain febrile for 2-3 days following empiric antibacterial coverage.

Hospital LOS following culture-proven onset of CBSI (post-CBSI HLOS) served as the primary end-point, with hospital costs representing the secondary endpoint. Inappropriate empiric therapy was defined as 1). treatment delay of ≥24 hours from candidemia onset with an antifungal agent active against the pathogen or 2). an inadequate dose of antifungal agent active against the pathogen. Adequacy of the dose was based on the dosages recommended by the Infectious Diseases Society of America and the individual antifungal package insert. The initial adequate dosage of fluconazole for susceptible isolates was defined as 6 mg/kg/day for *Candida albicans, Candida tropicalis*, and *Candida parapsilosis *in the face of normal renal function, and 3 mg/kg/day if creatinine clearance was < 50 ml/min. Fluconazole was not considered to be adequate at any dosage for *Candida krusei *or *Candida glabrata*. Total hospital costs (US$) represent the sum of costs across all individual hospital cost centers. These cost centers included room and board, pharmacy, radiology, and laboratory.

We performed descriptive statistics on demographic and clinical characteristics and outcomes, comparing those patients getting appropriate vs. inappropriate treatment. To assess the independent impact of inappropriate therapy on LOS and costs, we developed generalized linear models (GLM) using a gamma distribution for both the LOS and costs in order to compensate for their non-parametric distributions. Although only total costs were available rather than costs incurred following the development of the index infection, we arrived at CBSI attributable costs by adjusting for the HLOS prior to the CBSI onset. Statistical significance was set at the p ≤ 0.05.

All analyses were performed in Stata version 9.2 (Statacorp, College Station, TX).

## Results

Of the 135 ICU patients with candidemia within the specified time frame, 90 developed it within 14 days of hospital admission, 80 (88.9%) of whom received inappropriate treatment. There was no difference between the two groups based on age, gender, type and burden of comorbidities, or the severity of their acute illness (Table 1). Those patients receiving appropriate treatment were, however, more likely than the inappropriately treated group to be on a medial service (100% vs. 62.5%, p < 0.001), and to have a CBSI on admission (100% vs. 22.5%, p = 0.027).

**Table 1:** Baseline characteristics of ICU patients with Candida BSI

	Inappropriate (n = 80)	Appropriate (n = 10)	P value*
Age	57.2 ± 16.5	55.1 ± 21.8	0.774

Gender (Female)	38 (47.5%)	6 (60.0%)	0.518

Race (African American)	22 (27.5%)	4 (40.0%)	0.466

Medical service	50 (62.5%)	10 (100%)	0.027

CBSI on admission	18 (22.5%)	10 (100%)	<0.001

Comorbidities			

CHF	18 (22.5%)	3 (30.0%)	0.693

CAD	19 (23.8%)	4 (40.0%)	0.270

DM	25 (31.3%)	4 (40.0%)	0.721

COPD	16 (20.0%)	1 (10.0%)	0.680

Liver disease	4 (5.0%)	0	1.000

ESRD	7 (8.8%)	2 (20.0%)	0.261

HIV	1 (1.3%)	0	1.000

Cancer	23 (28.8%)	2 (20.0%)	0.720

Organ transplant	1 (1.3%)	1 (10.0%)	0.218

APACHE II score	16.3 ± 6.7	16.3 ± 7.8	0.990

MV	52 (65.0%)	6 (60.0%)	0.739

Pressors	23 (28.8%)	4 (40.0%)	0.479

CVC present	70 (87.5%)	8 (80.0%)	0.617

*P values derived using Student's t-test for continuous and Fisher's exact test for categorical variables

Microbiologically, there were no differences between the appropriately and inappropriately treated groups. In both the majority infections were due to *Candida albicans *(Table 2). The majority of patients were treated with empiric fluconazole, reflecting the approximately 80% fluconazole susceptibility among the isolates (Table 2). Among patients receiving inappropriate therapy, 95% was deemed inappropriate due to ≥24-hour delay in therapy, and in 26% this was due to an inadequate fluconazole dose.

**Table 2:** Microbiology and treatment parameters

	Inappropriate (n = 80)	Appropriate (n = 10)	P value
Microbiology*			

*C. albicans*	53 (66.3%)	5 (50.0%)	0.319

*C. glabrata*	13 (16.3%)	2 (20.0%)	0.671

*C. parapsillosis*	8 (10.0%)	2 (20.0%)	0.307

*C. tropicalis*	4 (5.0%)	1 (10.0%)	0.453

*C. kruzei*	2 (2.5%)	0	1.000

*C. lucitaniae*	1 (1.3%)	0	1.000

Fluconazole susceptible	63 (78.8%)	8 (80.0%)	1.000

Antifungal treatment¶			

Fluconazole	42 (52.5%)	6 (60.0%)	1.000

Caspofungin	35 (43.8%)	4 (40.0%)	

Amphotericin B	2 (2.5%)	0	

Voriconazole	1 (1.3%)	0	

Antifungal delayed 24+ hours	76 (95.0%)	0	<0.001

Inappropriate dose	21 (26.3%)	0	0.109

CVC removed	58 (82.9%)	6 (75.0%)	0.629

All p values are derived via Fisher's exact test

*Total number adds up to over 100% due to overlap in pathogens detected

¶Does not add up to 100% due to rounding

Unadjusted outcomes differed significantly between the two groups. The median hospital LOS was 13 days longer and the median hospital costs were nearly double in the group receiving inappropriate compared to appropriate treatment (Table 3). The differences in both crude hospital mortality (29% vs. 0%, p = 0.062) and post CBSI-onset LOS (13 vs. 8 days, p = 0.059) also favored appropriate therapy, though neither reached statistical significance at the p ≤ 0.05. In the adjusted analyses, inappropriate therapy for CBSI was associated with an incremental increase in the hospital LOS following CBSI onset of 7.7 days (95% confidence interval 0.6 to 13.5, p = 0.015) and an excess in hospital costs of $13,398 (95% confidence interval $1,060 to $26,736, p = 0.033) (Table 4).

**Table 3:** Unadjusted Outcomes

	Inappropriate (n = 80)	Appropriate (n = 10)	P value*
ICU LOS (days)			

Mean ± SD	10.8 ± 10.8	9.1 ± 10.1	

Median (IQR 25, 75)	8 (4, 14)	4 (2, 14)	0.371

Hospital LOS (days)			

Mean ± SD	24.4 ± 17.5	10.7 ± 9.4	

Median (IQR 25, 75)	21 (13, 28)	8 (3, 16)	0.002

ICU cost ($)			

Mean ± SD	$13,448 ± $14,872	$12,801 ± $14,324	

Median (IQR 25, 75)	$10,249 ($4,290, $18,757)	$5,545 ($2,750, $19,250)	0.559

Hospital cost ($)			

Mean ± SD	$66,584 ± $49,120	$33,526 ± $27,244	

Median (IQR 25, 75)	$52,437 ($35,977, $81,233)	$26,916 ($11,388, $46,454)	0.006

Hospital LOS (days) following onset of CBSI			

Mean ± SD	18.4 ± 17.0	10.7 ± 9.4	

Median (IQR 25, 75)	13 (8, 22)	8 (3, 16)	0.062

Hospital mortality	23 (28.8%)	0	0.059

*Derived using Mann Whitney U test for continuous variables and Fisher's exact test for categorical variable

**Table 4:** Utilization outcomes attributable to inappropriate antifungal treatment

Outcome	Point estimate	95% CI	P value
Excess hospital LOS (days) following onset of CBSI*	7.7	0.6-13.5	0.015

Excess hospital costs ($)†	$13,398	$1,060-$26,736	0.033

Based on generalized linear models with gamma distribution

*Generalized linear model, adjusted for all covariates with p < 0.2 in the univariate analysis plus age, APACHE II score, need for mechanical ventilation and need for pressors; manual backwards elimination to arrive at most parsimonious model

†Generalized linear model, adjusted for all covariates with p < 0.2 in the univariate analysis plus age and time from hospital admission to CBSI onset, APACHE II score, need for mechanical ventilation and need for pressors; manual backwards elimination to arrive at most parsimonious model

## Discussion

We have demonstrated that among ICU patients with CBSI onset within 14 days of admission, inappropriate therapy for CBSI is independently associated with an incremental increase of 8 days hospital LOS and excess hospital costs of over $13,000. In conjunction with previously reported data indicating that inappropriate therapy increases mortality, we confirm the compelling need for physicians to maintain a high index of suspicion for CBSI. Given that over 90% of the patients did not receive appropriate therapy, and the bulk of this was explained by a ≥24-hour delay in initiation of treatment, measures aimed at fostering earlier treatment along with education regarding the correct dosing of azoles may improve both clinical and economic outcomes.

Consistent with current epidemiologic data, *C. albicans *in our cohort was responsible for about 2/3 of all CBSI [4,5]. At the same time, the prevalence of azole resistant organisms was approximately 20%. Nevertheless, the vast majority of reasons for therapy being deemed inappropriate was not the selection of the wrong anti-fungal agent, but, rather, its delayed administration. This suggests that just by having a high index of suspicion for CBSI and instituting anti-fungal therapy while awaiting culture confirmation, 80% of the cases may be moved into the appropriate care category, and thus given the opportunity for better potential outcomes.

Historically, CBSI has been a syndrome with mortality in the range of 40%, and consequently, much work has focused on how to improve this outcome. Three studies have documented that prompt appropriate antifungal treatment independently decreases the risk for mortality. Morrell and coworkers demonstrated that a delay in treatment is frequent and one of as little as 12 hours from obtaining culture is independently associated with a 2-fold increase in the risk of hospital death [6]. Garey and coworkers confirmed these observations in a multicenter cohort, where they documented increasing mortality based on the delay, measured in days from when the culture was drawn to antifungal treatment [7]. Similarly, a recent study by Labelle and colleagues expanded the definition of inappropriate therapy for fungemia to encompass both delay of treatment and inadequate dosing of fluconazole [8]. Inappropriate therapy thus defined raised the risk of death 9-fold. All three of these studies underscore the importance of prompt appropriate treatment of candidemia in order to impact survival.

In addition to being deadly, candidal BSI remains a costly disease, with the cost of care ranging from $15,000-$40,000 per case [9-11]. In one report, the excess costs of care for candidemia were more than $15,000 over the costs related to treating BSIs due to bacterial pathogens [9]. Driving these greater costs was an accompanying 5-day excess length of stay in the setting of candidemia. Our study builds on these observations and extends them to quantify excess LOS and costs associated with inappropriate treatment of BSI, calling into question whether more prompt identification and treatment of CBSI might not only improve survival, but also reduce expenses. A major obstacle to this in clinical practice remains the lack of adequately validated and sufficiently specific predictive tools to determine the likelihood of CBSI in a critically ill patient with multiple risk factors [12-14]. Therefore, much is left to clinical judgment, particularly in terms of evaluating the balance between an individual patient's risk for CBSI and the overall risk for developing resistance to anti-fungal agents due to their overuse.

Our observations echo those reported in other syndromes. For example, Shorr and colleagues evaluated the economic implications of instituting an early goal-directed therapy protocol in the emergency department of an urban tertiary care hospital [15]. In this study, appropriate treatment was associated not only with reduced mortality [16], but also with a reduced LOS and hospital costs. Similarly, the same group reported that inappropriate treatment of methicillin-resistant *Staphylococcus aureus *(MRSA) sterile site infections was associated with a prolongation of hospital LOS and an increase in costs [17].

Our study is subject to a number of limitations. Its retrospective design may have predisposed it to a selection bias. We attempted to mitigate this by enrolling consecutive patients meeting the pre-specified enrollment criteria. Although confounding is a potential problem with all cohort studies, we adjusted our analyses for potential covariates. Nevertheless, the possibility of residual confounding persists. The study's single center nature may limit the results' generalizability to centers similar to ours.

Despite these limitations, to the best of our knowledge, this is the first study to quantify the potential excess costs related to inappropriate treatment of CBSI. Together with the evidence for improved survival with appropriate treatment and the low prevalence of such, our results suggest a need for a more balanced approach to CBSI identification and treatment. Not only is there a need to heighten clinical suspicion among appropriate patient populations, but there is a clear need for rapid bedside tools to identify this infection, so as to commence prompt therapy. Given the lack of success developing a prediction model, clinical judgment could be aided greatly by a rapid bedside assay, such as β-D-glucan, though this has not been validated among the critically ill [18,19].

## Conclusions

In summary, the current data shed light on the potential cost and hospital resource savings that may be possible if CBSI were to be treated promptly and appropriately. This information, in conjunction with the life-saving potential of such a strategy, makes a compelling argument for improving processes around identification and treatment of this syndrome in critically ill patients.

## Key messages

• Inappropriate treatment of candidemia in the ICU is frequent.

• Having a high index of suspicion for CBSI and instituting anti-fungal therapy while awaiting culture confirmation may result in moving 80% of the cases from the inappropriate into appropriate care category.

• Inappropriate therapy for CBSI is independently associated with a significant increase in hospital resource utilization.

## Abbreviations

BSI: blood stream infection; LOS: length of stay; BJH: Barnes Jewish Hospital; ICU: intensive care unit; CBSI: Candida blood stream infection; HLOS: hospital length of stay; GLM: generalized linear model; MRSA: methicillin-resistant *Staphylococcus aureus*; CHF: congestive heart failure; CAD: coronary artery disease; DM: diabetes mellitus; COPD: chronic obstructive pulmonary disease; ESRD: end-stage renal disease; HIV: human immunodeficiency virus; CVC: central venous catheter; MV: mechanical ventilation.

## Competing interests

This study was funded by a grant from Astellas Pharma US, Inc., Deerfield, IL, the manufacturer of micafungin.

## Authors' contributions

MZ contributed to study conception and design, and analysis and interpretation of data; was involved in drafting the manuscript and revising it critically for important intellectual content. MK contributed to study conception and design, acquisition of data, and interpretation of data; was involved in revising the manuscript critically for important intellectual content; HA contributed to conception and design, and acquisition of data; was involved in drafting the manuscript; AL made substantial contributions to conception and design, acquisition and interpretation of data; was involved in revising the manuscript critically for important intellectual content; SM contributed to conception and design, and acquisition of data; was involved in revising the manuscript critically for important intellectual content; SK made substantial contributions to conception and design of the study; was involved in revising the manuscript critically for important intellectual content; AF have made substantial contributions to conception and design, analysis and interpretation of data; was involved in revising the manuscript critically for important intellectual content; All authors gave final approval of the version to be published.

## Pre-publication history

The pre-publication history for this paper can be accessed here:

http://www.biomedcentral.com/1471-2334/10/150/prepub
